# Research for Actionable Policies: implementation science priorities to scale up non–communicable disease interventions in Kenya

**DOI:** 10.7189/jogh.07.010204

**Published:** 2017-06

**Authors:** Sujha Subramanian, Joseph Kibachio, Sonja Hoover, Patrick Edwards, Evans Amukoye, Mary Amuyunzu–Nyamongo, Gisela Abbam, Naftali Busakhala, Abigail Chakava, Jonathan Dick, Robai Gakunga, Gladwell Gathecha, Rainer Hilscher, Muhammad Jami Husain, Lydia Kaduka, James Kayima, Alfred Karagu, Dorcas Kiptui, Anne Korir, Nkatha Meme, Breda Munoz, Walter Mwanda, Daniel Mwai, Julius Mwangi, Esther Munyoro, Zachary Muriuki, James Njoroge, Elijah Ogola, Carol Olale, Deborah Olwal–Modi, Rose Rao, Saras Rosin, Onyango Sangoro, Daniel von Rège, David Wata, Pam Williams, Gerald Yonga, Participants from the NCD Symposium in Kenya

**Affiliations:** 1 RTI International, Waltham, Massachusetts USA; 2 Kenya Ministry of Health, Nairobi, Kenya; 3 Kenya Medical Research Institute, Nairobi, Kenya; 4 Africa Institute for Health and Development, Nairobi, Kenya; 5 GE Healthcare, Chalfont St Giles, UK; 6 Global Diagnostic Imaging, Healthcare IT & Radiation Therapy Trade Association, Arlington, Virginia, USA; 7 Moi University, Academic Model Providing Access to Healthcare, Eldoret, Kenya; 8 Novo Nordisk A/S, Copenhagen, Denmark; 9 Independent Consultant, Nairobi, Kenya; 10 US Centers for Disease Control and Prevention, Atlanta, Georgia, USA; 11 Makerere University, Uganda Heart Institute, Kampala, Uganda; 12 Nairobi City County, NCD Unit, Nairobi, Kenya; 13 University of Nairobi, Nairobi, Kenya; 14 Kenyatta National Hospital, Nairobi, Kenya; 15 National Hospital Insurance Fund, Nairobi, Kenya; 16 Danish Embassy, Nairobi, Kenya; 17 Kenya Cancer Association, Nairobi, Kenya; 18 AstraZeneca Kenya, Nairobi, Kenya; 19 Stockholm Environmental Institute, Nairobi, Kenya; 20 Médecins Sans Frontières, Belgium, Nairobi, Kenya; 21 NCD Alliance Kenya, Nairobi, Kenya

Low– to middle–income countries (LMICs) are disproportionately affected by the rise in prevalence of non–communicable diseases (NCDs). According to the World Health Organization, four groups of diseases– cardiovascular disease, cancer, respiratory disease and diabetes – comprise 82% of NCD deaths worldwide and three–quarters of the deaths related to NCD occur in LMIC [1]. In Sub–Saharan Africa, the World Bank estimates that nearly 46% of all deaths will be attributable to NCDs by 2030, and 41% of all deaths for those aged 15–59 will be due to NCDs [2].

Similar to other Sub–Saharan African countries, Kenya is experiencing an explosive growth in NCDs, especially those related to cancer and cardiovascular diseases [3]. There is therefore an urgent need to determine implementable interventions to reduce the growing burden from these and other NCDs, including respiratory diseases, injuries and mental health. Although there are many ongoing research studies and demonstration programs [4], it is not clear whether these activities address the Kenyan Government’s evidence needs and priorities to support their NCD strategy [5]. To understand the current research landscape in order to guide the implementation research agenda in Kenya, RTI International, Kenya Ministry of Health, NCD Alliance Kenya, Kenya Medical Research Institute, and University of Nairobi hosted a two–day symposium on September 7–8, 2016 in Nairobi, Kenya. The symposium was entitled, “Research for Actionable Policies: Implementation Science Priorities to Scale Up Non–Communicable Disease Interventions in Kenya.” The sections that follow provide an overview of the meeting including its purpose and objectives, a summary of the proceedings and recommendations to address gaps in NCD implementation science research in Kenya.

## SYMPOSIUM OBJECTIVES AND STRUCTURE

The symposium addressed three specific objectives. First, we wanted to catalog promising and innovative intervention strategies applicable to the Kenyan setting in order to account for existing programs and ongoing implementation research activities. Next, we endeavored to identify gaps in research to support implementation of cost–effective interventions and assess areas where research capacity is needed. Lastly, the symposium participants generated recommendations to create a road map for implementation science research to advance evidence–based NCD prevention and control in Kenya. The symposium attempted to address a broad range of NCDs but its main focus was on cardiovascular diseases and cancers as these two diseases pose the highest burden.

Representatives from the Kenya’s Ministry of Health opened the symposium by setting the framework for the meeting and outlining the importance of implementation science. The US National Institutes of Health defines implementation science as the study of methods to promote the integration of research findings and evidence into health care policy and practice. The key challenge posed to the participants was to consider whether the current implementation science research conducted in Kenya supported the government’s NCD strategy [5], and if the research did not, how could the country move toward using health research to inform policy and practice.

The two–day symposium consisted of 14 sessions [6]. During the first day, stakeholders presented on the burden of NCDs internationally, in Sub–Saharan Africa and in Eastern Africa with a focus on Kenya. They also discussed the role of research and strategic planning to combat them. Toward the end of day one, participants convened in small groups to discuss gaps in NCD implementation science research and brainstorm ideas to address the gaps. On the second day, panel members presented and discussed program implementation at the community level and through public–private partnerships. The symposium closed with a panel discussing lessons learned from the two days and summarizing specific recommendations to guide future implementation science research in Kenya. Approximately 100 individuals representing multiple disciplines from research institutes, civil society, governmental organizations, health care providers, industry and international organizations participated during each day of the symposium.

## OVERVIEW OF THE PROCEEDINGS

### Burden of NCDs – challenges and opportunities

#### Risk factors

NCDs account for 27% of all deaths in Kenya, totaling almost 100 000 people per year [7]. Additionally, NCDs contribute to over 50% of inpatient admissions and 40% of hospital deaths and therefore have a substantial impact on health care budgets [7]. Data from the 2015 Kenya StepWise Survey indicates that approximately 8% of the Kenyan population smoked daily, between one–fifth and one–quarter were exposed to second hand smoke, 17% of men and 35% of women were overweight or obese and nearly one–quarter of the population had high blood pressure; all of these risk factors and conditions are contributing risk factors to NCDs [8]. These data support the urgent need to have implementable interventions to reduce the projected high burden of NCDs in Kenya. In subsequent presentations, the NCD leaders from the Tanzanian and Ugandan Ministries of Health and other researchers acknowledged that NCD trends in additional East African countries were overall similar to those reported for Kenya. Further, the symposium participants concluded that the challenges of implementing health care interventions along the continuum of care had many similarities across these countries, although interventions would need to be tailored to ensure optimal integration with the existing health care system and infrastructure in each country.

#### Current initiatives to address policy gaps

In 2015, Kenya launched the National Strategy for the Prevention and Control of NCDs (2015–2020) to reduce NCD mortality by 25 percent by 2025 in accordance with World Health Organization’s (WHO) recommendations [5]. In addition, Kenya has also developed a National Diabetes Strategy (2010–2015) and a National Cancer Control Strategy (2011–2016) [9,10] which are intended to raise patient awareness and spur initiatives to improve management infrastructure. Furthermore, to address key risk factors for NCDs, Kenya has developed policies and regulations to address tobacco use and excessive alcohol consumption [11]. The Tobacco Control Act of 2007 is the principal law governing tobacco use in Kenya. This comprehensive law defines key terms and covers topics including restrictions on public smoking; tobacco advertising, promotion and sponsorship; and packaging and labeling of tobacco products. The Tobacco Control Regulations of 2014 further regulates selected provisions under the Tobacco Control Act including public smoking restrictions, tobacco product and tobacco industry disclosures, which came into effect in September 2016. Currently, there is a 49% tax on the sale price of tobacco products. In 2010, the Alcoholics Drinks Act was enacted by the Kenyan parliament. It has not yet been fully implemented nationwide as devolution, which was adopted in 2013, shifted decisions about whether to adopt and implement the law to Kenya’s 47 county governments.

Overall, Kenya has made efforts to generate policies through legislation and increase knowledge through research on tobacco and alcohol control [12-14]. Clearly more needs to be done but there are other areas related to diet, specifically salt and sugar consumption, which currently have no legislation nor large–scale research initiatives. High levels of obesity, especially among women, and hypertension are the main risk conditions for NCDs in Kenya and therefore addressing the underlying risk factors could have a substantial impact on reducing the burden from NCDs. Salt reduction is one of the most cost–effective interventions and implementing public awareness programs using mass media on diet and physical activity is classified as a ‘best buy’ (or best practice) [15,16]. Research is required to identify optimal approaches that should be adopted in the Kenyan context to ensure policies are successful when implemented.

Evidence from other countries points out that successful implementation of targeted policies can lead to reduction in harmful risk factors and risk conditions [15,16]. An overview presentation from the US Centers for Disease Control and Prevention provided several examples on policy approaches to successfully address NCDs that have been applied in the United States [17,18]. These include defining national targets, such as the decades–long Healthy People initiatives, promoting multisector NCD action plans, implementing policies to address NCD risk factors and ensuring appropriate treatment when required. High impact successful initiatives have included national, state and local level policies and legislations on tobacco control, enforcements of alcohol levels to reduce road traffic accidents and initiatives to reduce sodium content in the food supply.

#### Economic burden and finances

Given that NCDs are debilitating and long–lasting, they are often expensive to treat. And, as NCDs continue to affect younger populations, future productivity is imperiled. According to survey findings from Kenya, NCDs are associated with a 33.2% reduction in household income [19]. Further, in comparison with households with other chronic conditions, those with NCDs had 26.1% lower average income. Additionally, there was an inverse relationship between education and the presence of NCDs, which highlights the potential for NCDs to increase the disparities already experienced by those who are less affluent.

Only a small percentage of Kenyans have health insurance and participants offered a number of suggestions to reduce the economic burden from NCDs [20]. The National Hospital Insurance Fund (NHIF) is one possibility to assist Kenyans diagnosed with NCDs. The program collects a percentage of employees’ salary and places it into a risk pool, and participants can select various benefit packages, which include inpatient and other comprehensive selections. The government of Kenya has designated the NHIF as the driver of its approach to universal health coverage and anticipates that increasing percentages of the population will be covered under various schemes over time. Because of the relationship between education and NCDs, symposium participants also suggested that public education be improved with an increase in country–wide NCD awareness campaigns and affordable preventive health care services.

### Evidence–based strategic planning to address NCDs in Kenya

#### Model–based prioritization of interventions

Researchers from Kenya’s Ministry of Health and RTI International spoke to the importance of strategic planning when addressing NCDs in Kenya. First, it was necessary for the country to assess the burden of NCDs and consider the consequences of inaction. Second, the country should assess the effectiveness of primary prevention (eg, immunizations, education) and secondary prevention (eg, screening and treatment, wellness care). Third, Kenya needs to examine the cost of implementation and determine the optimal interventions to target in the short–term, medium–term and long–term timeframes. This systematic approach will also identify the gaps in the research and data sources that need to be addressed to inform future strategic planning.

RTI scientists described qualitative and quantitative tools used to inform strategic planning, including economic modeling to simulate the impact of interventions. For example, population–attributable risk models developed by RTI researchers indicated that if tobacco were no longer a factor in individuals’ behaviors (ie, if people no longer smoked), deaths attributed to cardiovascular disease (CVD) and cancer would be reduced by 9% by 2030 (compared to not doing anything); if high blood pressure was no longer a risk factor, deaths attributed to CVD would be reduced by 33% by 2030 (compared to not doing anything) [21].

Additionally, in operationalizing Kenya’s NCD Strategic Plan, RTI researchers reiterated the importance of considering the cost of implementing interventions alongside the projected benefits to ensure interventions are selected to achieve maximum impact [22]. The preliminary research presented indicated that women’s health can be improved by reducing obesity through diet and physical activity interventions; taxes applied to tobacco and alcohol will have a greater impact on the health of men than women because of the existing patterns of smoking; and management of hypertension is essential but potentially expensive to provide on the scale required, so primary prevention should be intensively pursued.

#### Data needs for planning and evaluating NCD interventions

Several presenters focused on the benefits and challenges of collecting data to monitor NCDs. Benefits included understanding NCD patterns, conducting research reflective of the data, and supporting health departments in guiding and planning disease prevention and control programs. Currently, there is limited data on NCDs to monitor and track the impact of interventions, but new data initiatives are in various stages of planning and implementation. Kenya Medical Research Institute (KEMRI) staff presented plans to expand cancer registration in Kenya to create a National Cancer Registry and a pilot study is under way to evaluate opportunities to create a stroke registry [23]. However, several challenges remain, including lack of staff at public health institutions to ensure high quality data collection at the point of service delivery, lack of continuity of funding and reliance on manual data collection and reporting [24]. The Academic Model Providing Access to Healthcare (AMPATH) has developed an electronic health information system that has been successfully implemented in Western Kenya and this approach could provide important lessons for other institutions to adopt comprehensive electronic health information technology systems [25,26].

#### National– vs county–level planning

Several presenters discussed experiences in NCD planning at both the national and county level. Presenters noted the necessity for Kenya to improve its health infrastructure due to the loss of health tourism dollars to India, South Africa and the United States. They argued that maintaining these resources within Kenya will allow for investment in additional health care facilities. Although Kenya does have two public hospitals to which patients from Tanzania and Uganda travel to, these hospitals cannot meet overall demand and there is a need to offer competitive pricing. Tertiary level hospitals can only be established in specific locations and therefore coordination between the national and county level governments is critical to provide NCD services across the continuum of care.

A Nairobi City Country representative described the establishment of an NCD Unit in the County Health Office in response to a key objective in the County’s Health Sector Strategic and Investment Plan 2013–2018. The goals of the unit include improving awareness of all NCD risk factors, strengthening screening programs and treatment for all NCDs, and developing specialized clinics with sustainable services. As a result of the unit, to date, there has been improvements in NCD service delivery but there have been challenges as well, including limited funding, poor data collection and the need to address multiple risk factors, such as environmental pollution.

### NCD clinical– and community–level implementation

#### Integration of service delivery

Several models of integrating service delivery were presented. The program at AMPATH provides integrated services including breast and cervical cancer screening. The cervical cancer screening program is well established while the breast cancer screening initiative using clinical breast exam is relatively new. A recent study found that those with prior knowledge of breast cancer risk factors are more likely to participate in screening [27]. A key feature at AMPATH is the focus on increasing linkages with patients and retaining individuals so they stay engaged and receive continual services. There are ongoing plans to track patients using common electronic medical records (EMR) and universal identification across health centers to monitor success of integrated delivery and retention.

**Figure:**
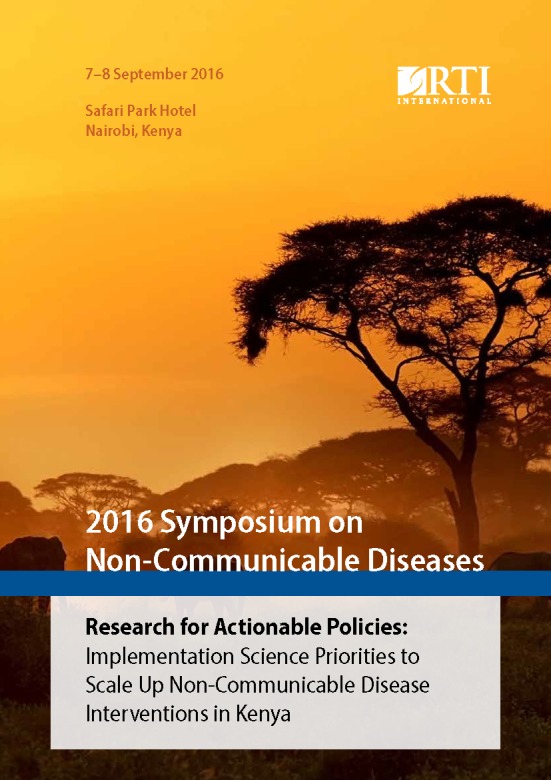
Photo: 2016 Symposium on Non–Communicable Diseases (front page of the conference program)

Representatives from MSF Belgium described a low–cost approach to integrated NCD care using simplified treatment and referral guidelines and a one–stop clinic location for all basic services. The patient–focused approach is supported through task shifting to allow NCD patients to be seen on all clinic days and ‘Adherence Management Clubs’ have been established where HIV and NCD patients meet in groups to facilitate compliance with medications. NCDs are chronic diseases by nature and thus pose ongoing challenges related to patient retention and long–term disease management.

Additionally, the community–based hypertension management under AstraZeneca’s Healthy Heart Africa program aims to increase capacity through training of health care workers and improvement of the medication supply chain. Further discussions were also focused on leveraging the HIV platform to offer NCD services. AstraZeneca and the United States President's Emergency Plan for AIDS Relief (PEPFAR) have recently announced a pilot initiative in Kenya which will provide an opportunity to use hypertension as an entry point to provide HIV services to a key population of younger working age males (25–50 years) and, alternatively, hypertension services to a large cohort of individuals through the HIV/AIDS infrastructure developed by PEPFAR. Integration of health services is not without its challenges and a recent study in Kenya which found resistance among health care workers to incorporate NCDs into HIV programs due to the perceived substantial increased workload. Similar findings related to workforce related barriers to integration have also been reported in Uganda and Tanzania [28,29] and therefore these challenges to integrated service delivery need to be a focus of ongoing implementation research.

#### Infrastructure and capacity building

A multidisciplinary team of researchers presented on the need to increase staff capacity and their ability to provide all services related to cancer screening programs. Statistics presented showed that Kenya is understaffed in numerous key provider categories including oncologists, radiation therapists and oncology nurses [30]. Participants stressed the need for better infrastructure in order to increase access to diagnostic and treatment services, which are the cornerstone of any successful screening program. A critical need identified was the improvement of laboratory capacity in Kenya which was described as one of the weakest links in the current health care system. A World Bank–supported demonstration project is establishing a network of laboratories to increase access to diagnostic services for chronic diseases and NCDs. Other key areas for improvement are palliative services and hospice care, which are both inadequate to meet the current patient needs.

#### Patient awareness and barriers to cancer care

Researchers in many of the sessions noted that not all Kenyans were familiar with NCDs, and even when aware, very few have been screened for NCDs. For example, results from an analysis of the 2014 Kenya Demographics and Health Survey indicated that 76% had heard of cervical cancer, while only 14% of women have been screened, and only 65% of men had heard of prostate cancer, with only 3% having been screened [20]. One of the presenters provided a framework on how to utilize health communication science to change patient behavior for NCD prevention and control, which included educating patients about how their behavior contributes to risk factors and motivating them to be screened. A recent patient survey at Kenyatta Hospital showed that almost a third of the patients had missed or delayed treatment mainly due to financial reasons, problems with getting transportation for treatment, and lack of accommodations once in Nairobi. Increasing patient awareness is important but there are additional barriers that need to be addressed to ensure optimal delivery of services along the continuum of care.

#### Public–private partnerships

Government funding of screening, diagnosis and treatment programs can be enhanced with partnerships with private entities to fill gaps in health care service delivery. A panel from the pharmaceutical and diagnostic industry spoke to their companies’ programs and partnerships in Africa and in Kenya specifically. Examples of activities partners conducted included training of health care staff and providers about diagnosing and treating NCDs and simplifying treatment protocols for adoption. Many of the companies were committed to increasing access to prescription drugs for patients and offered treatments at reduced or affordable prices. Companies also worked with local civil society to raise awareness of NCDs and improve skills in outreach, advocacy and fundraising. Novo Nordisk also presented an innovative approach through their Base of Pyramid project to collect and analyze data for evaluation and program improvement. Coordination of activities between the private and public sectors to ensure synergies and to reduce duplication was indicated as key aspect in discussion sessions.

## SUMMARY OF KEY RESEARCH RECOMMENDATIONS

Several critical research gaps were identified during group discussion, panel presentations and participant dialogue. The research roadmap for Kenya should include the following recommendations.

1. Evaluate optimal approaches to implement policies for primary and secondary prevention tailored to the Kenyan setting:
  - There is lack of systematic knowledge on dietary patterns and individual food purchase decisions. Policies to reduce salt consumption, which has shown to reduce high blood pressure and is a WHO ‘best buy’ intervention, should be implemented in Kenya after research on optimal implementation strategies is conducted. Additionally, obesity is a substantial risk factor for NCDs, especially among women, and interventions to reduce sugar consumption should be systematically evaluated.
  - Interlinkages between infections and NCDs, especially cancers, is well established and adequate research into scale up of vaccination programs to reduce targeted infections is required (especially Human Papillomavirus vaccine to substantially decrease the incidence of cervical cancer).
  - It is also important to assess and understand the life course perspective as many of the risk factors for NCDs can be present at birth or acquired during early childhood (such as stunting which increases risks of nutrition–related chronic diseases including diabetes, hypertension, and obesity in the future).
  - Given resource constraints, it is essential to understand which cost–effective interventions are most affordable in the Kenyan context to operationalize national NCD strategic plans.
2. Generate evidence–based health communication messaging for all key stakeholders and strengthen community participation:
  - There is a lack of understanding of the patients’ perspective and their health seeking behavior. A systematic evaluation of the social determinants of the use of preventive services is required.
  - Research is needed to determine the best approaches to communicate risk factors to patients and communities. Providers need to be engaged as well given the vital role of patient–provider communication in increasing use of NCD preventive services.
  - Additionally, improved approaches to communicate with policy makers is necessary to appropriately and successfully advocate for NCDs.
3. Assess capacity to deliver NCD services along the continuum of care:
  - A systematic evaluation of infrastructure investments that are required to provide optimal oncology diagnostic and treatment services should be initiated. Screening programs for breast and cervical cancer cannot be scaled up without adequate provision of services along the continuum of care for appropriate triage for diagnosis and treatment.
  - Provider training needs should be evaluated from the primary level to the specialty care setting. Overall, health services delivery is still geared toward addressing communicable diseases and there needs to be a shift to dual care for communicable and non–communicable diseases.
  - Better understanding of task shifting and task sharing is needed to ensure NCD program implementation leads to successful scaling up of services. Integration with other successful programs, such as maternal and child health services, should be evaluated to assess efficiency in NCD service delivery.
4. Ensure the NCD planning process is evidence–based and data–driven through targeted data collection efforts:
  - Estimates of regional data are not accurate or nonexistent; this information is needed to evaluate the extent to which NCD policies and interventions are generalizable across regions in Kenya.
  - Opportunities need to be explored that piggyback NCD–related data elements onto existing data collection mechanisms and encourage standardized data collection at the county level.
  - Support should be provided to ensure sustainability of the newly–launched National Cancer Registry and planned establishment of the stroke registry.
5. Investigate innovative approaches to finance NCD services along the continuum of care:
  - The National Health Insurance Fund is evaluating coverage for cancer screening; stakeholder engagement is needed to ensure that the preventive package of coverage is comprehensive and appropriate for the Kenyan setting.
  - The role of private insurance and other risk–based pooling of resources needs to be evaluated.
  - Cost versus quality of coverage in the public versus private sector should be evaluated to provide health consumers with more transparent information for decision making and facility selection.

Additional recommendations were also shared on how to address the research gaps identified. The steps include:

1. Advocating for a systematic process to develop research capacity across Kenyan institutions so researchers can contribute towards addressing the identified gaps. Initiatives could include fellowships, hands–on mentoring and other training programs.
2. Leveraging existing data sources from national and local surveys and studies to get a deeper understanding of NCD risk factors and the underlying social determinants.
3. Engaging in public–private partnerships to enhance resource sharing and address common goals of increasing awareness of NCD risk factors and availability of screening, diagnosis and treatment services.
4. Collaborating to seek strategic funding for new research studies that avoid replication to ensure efficient use of limited resources.
5. Enabling deeper regional cooperation across East African countries to allow for the sharing of lessons learned, as much of the underlying patient and health system factors are similar.

## CONCLUSIONS

The NCD–related research recommendations proposed in this report will require resources to implement, but the cost of inaction is extremely high [31]. Partnerships and multilevel coordination are essential to ensure available funding is maximized and duplication is avoided. The recommendations from this symposium can serve as a road map to guide the future research investments that have to be made to support the planning and implementation of policies and interventions that are likely to yield maximum benefits to Kenyan society. Implementation cannot wait until all the evidence is gathered, as the needs are immediate and pose a high burden to individuals, communities and the nation as a whole. Targeted pilot programs can serve as a vehicle to test strategies on a limited scale and lessons learned can then guide widespread implementation. The county–level organizations and the national government will need to coordinate implementation activities to ensure that an optimal learning environment is created where evidence generated in one county can be disseminated to improve the health of all Kenyans.
